# Optimising interpreter mediated dementia assessments

**DOI:** 10.1002/alz70857_096670

**Published:** 2025-12-24

**Authors:** Naheed Mukadam, Renelle Bourdage

**Affiliations:** ^1^ University College London, London, London, United Kingdom; ^2^ Erasmus University Medical Center, Rotterdam, South Holland, Netherlands

## Abstract

**Background:**

Many older people from ethnically diverse groups across European countries experience cognitive decline and dementia. With increasing diversity of older adults presenting to cognitive assessment services, there is an urgent need to improve the quality of assessment provided to such patientsso that dementia and other neuro‐cognitive disorders can be diagnosed in an accurate and timely manner. Many of these assessments will necessitate use of interpreters but quality of these assessments varies and knowledge of dementia among interpretersisinconsistent. An intervention recently trialled in Australia found that an interpreter training intervention improved knowledge of dementia among interpreters. We aim to build on this successful intervention by translating and adapting it for use in seven European countries

**Method:**

We will complete the following work packages: 1. Review existing evidence including the Australian intervention and other similar interventionsto identify core components and good practice. 2. Recruit and engage ∼40 stakeholdersincluding patients, service managers, clinicians, and interpreters about experiences using interpreter‐mediated cognitive assessment. 3. Translate and adapt the intervention for use in seven different European countries and modify in accordance with feedback. 4. Pilot the intervention with interpreters and modify based on feedback 5. Identify future funding to implement and assessthe training programme across Europe.

**Result:**

We will present results from qualitative interviews conducted across seven European countries to highlight disparities in use of interpreters in dementia diagnostic and assessment services and identify how thes processes could be improved.

**Conclusion:**

Use of interpreters is often crucial to accurate diagnostic assessment when assessing diverse groups for cognitive impairment. This programme of works aims to highlight how this process can be improved across Europe with the aim of producing interpreter training to enhance skills in this area.

